# Water security in Mbagathi and Stony Athi catchments within Kenya’s SGR corridor under environmental and socio-economic change

**DOI:** 10.1007/s43832-025-00251-9

**Published:** 2025-07-09

**Authors:** Catherine C. Sang, Daniel O. Olago

**Affiliations:** 1 Department of Environmental Planning, Sustainability and Geoinformatics, University of Eldoret, 30100 Eldoret, Kenya; 2 Department of Earth and Climate Sciences, Institute for Climate Change and Adaptation, University of Nairobi, 00100 Nairobi, Kenya

**Keywords:** Water demand, Urbanization, Land use changes, Climate change, Infrastructure development, Sustainable water management

## Abstract

Water insecurity, driven by urbanization, population growth, land use and climate change, poses a global challenge. This study examines water supply and demand trends in the Mbagathi-Stony Athi sub-catchments, highlighting urbanization's impact in a semi-arid context. Using GIS, and the WEAP model, various scenarios were simulated. Results show annual rainfall increased insignificantly (*p* = 0.61) from 1981 to 2019. By 2063, rainfall is projected to rise by 12.43% (RCP 4.5) and 21.02% (RCP 8.5). Mean temperature increased by 0.88 °C (1981–2019) and is projected to rise by 1.70 °C (RCP 4.5) and 1.75 °C (RCP 8.5) by 2063. Land use analysis (2000–2019) showed a 53.67% increase in built-up areas and a 99.32% decline in wetlands. Between 2000 and 2019, the annual supply, demand, and unmet demand increased by 171.64%, 147.56%, and 73%, respectively. Land use changes between 2000 and 2019, particularly the increase in shrublands and decline in bare land, contributed to a 25.51% decrease in surface runoff and a 3.55% rise in total annual evapotranspiration. Future projections indicate surface runoff decreases of up to 4.47% under RCP 4.5 and increases of 9.38% under RCP 8.5. Potential evapotranspiration is projected to rise by 23.39% (reference), 16.44% (RCP 4.5), and 11.19% (RCP 8.5). Water demand will increase across all scenarios, peaking at 184% under high urbanization, while unmet demand will rise by up to 162.47% under irrigation expansion. Water scarcity is expected to worsen due to climate change, population growth, and land use shifts. These findings inform sustainable water resource management in development corridors.

## Introduction

Water security entails ensuring sustainable access to adequate quantities of good-quality water for various purposes, from human well-being and socio-economic development to safeguarding ecosystems and peace [1, 2]. The primary threat to water security is water scarcity, driven by natural and human factors [3]. By 2010, around 80% of the global population faced significant water security challenges, with 1.7 billion people in chronic high water-scarce conditions [4]. Currently, more than 2.3 billion people reside in water-stressed regions, and 3.6 billion experience water scarcity for at least one month annually. This number is projected to reach 4.8–5.7 billion by 2050 without interventions [5]. Water scarcity adversely affects agriculture, food security, livelihoods, economies, and ecosystems [6–9] and is expected to worsen due to factors like economic development, rapid population growth, infrastructure expansion, intensified agriculture, pollution, environmental degradation, land use changes, and climate change, exacerbated by inadequate water resource management [10–12]. Water demand has outpaced supply [13, 14], which is deteriorating in both quality and quantity due to climate change, hydrologic variability, and environmental degradation [15]. Approximately 26% of the world's population, or 2 billion people, lack access to safe water, with 733 million living in high or critically water-stressed countries [16]. The most affected are often marginalized communities in underserved areas [17].

Development corridors, designated areas for stimulating economic growth, have driven urbanization, industrialization, infrastructure improvements, and land use changes [18–20]. More generally, land use changes driven by economic development and accelerated population growth influence the hydrological cycle components [21–23], resulting in water pollution, quantity reduction, and ecosystem degradation [10, 24–28]. However, this rapid development, coupled with climate change, escalating water demand due to economic and population growth [15, 29], and failure to account for the sustainability of available water resources in most planning and water use [15], has amplified water insecurity. Further, this disproportionately affects Africa due to its high population growth rate (2.2% per year compared to the global average of 1.2% per year) [30].

In East Africa, temperature projections indicate a rise of 0.9–3.4 °C by the 2060s and 1.3–5.5 °C by the 2090s, leading to increased evaporative demand [31]. Rainfall patterns are less certain in terms of magnitude and direction of change, but there are indications of an overall increase [31]. Kenya grapples with water scarcity, which hampers economic growth [32, 33]. With an annual per capita available water resource of less than 500 m^3^/c/year, the country is classified as water-scarce [33]. Kenya’s water resources face ongoing challenges due to the impacts of climate change and human activities [34–37]. Presently, water coverage in Kenya stands at 60%, with a substantial 45% of non-revenue water [38]. If the current water management approach persists, the country may face a significant 30% gap between available freshwater supply and demand by 2030 [39]. To achieve water security in these regions, it's imperative to address climate change impacts and uncertainties through robust and evidence-based water resource management strategies.

In the case of the Standard Gauge Railway (SGR) corridor, most of the areas it traverses are classified as drylands, making them highly susceptible to frequent and prolonged droughts [40, 41]. Additionally, the corridor passes through Nairobi County, Kenya’s [42, 43]. The two catchments under study—Mbagathi and Stony Athi Rivers—are particularly affected by drought, further exacerbating water scarcity in the region ([44]. Moreover, these rivers experience high levels of pollution due to their proximity to industrial zones, densely populated urban centers, and towns along the corridor [45]. Soil erosion in some areas contributes to sedimentation, degrading water quality and affecting aquatic ecosystems [46]. Furthermore, there is significant over-extraction of water from these rivers for domestic use, irrigation, and other economic activities, further straining the already limited water resources [47]. These factors highlight the critical water-related challenges within the SGR corridor, emphasizing the need for sustainable management strategies.

Despite the growing awareness of water insecurity and its drivers, there remains a gap in localized, integrated assessments that examine how combined factors such as climate change, population growth, land use change, and economic development influence water availability—particularly within strategic development corridors like the SGR. Moreover, few studies employ scenario-based tools to evaluate future risks and support adaptive planning for water resource sustainability in dryland river catchments that are undergoing rapid development, facing environmental stressors, and are highly vulnerable to over-extraction, pollution, and drought. This lack of targeted, evidence-based planning limits the effectiveness of policy and intervention efforts.

To address this gap, this study assesses the current water supply and demand situation in the Mbagathi and Stony-Athi River catchments—located along Kenya’s Standard Gauge Railway corridor—and evaluates the potential impacts of future climate, demographic, and development scenarios on water availability. Using the Water Evaluation and Planning (WEAP) model, the study simulates the effects of climate change, land use transitions, economic development, and population growth on local water resources. The findings aim to inform sustainable water management strategies and provide insights that can be applied to other regions facing similar challenges.

## Materials and methods

### The study area

**Fig. 1:**
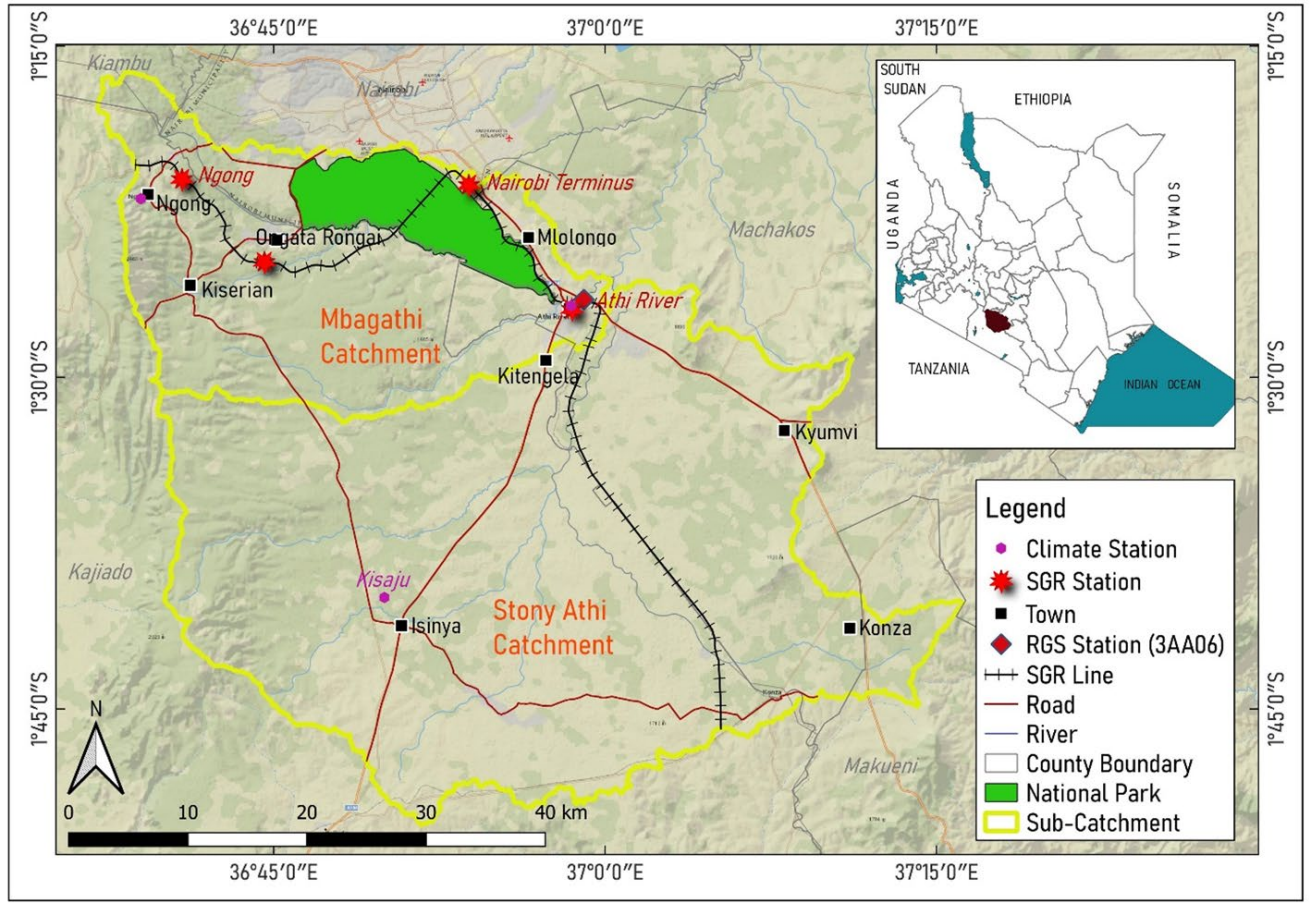
Location of the Stony Athi and Mbagathi sub-catchments

The study area comprises the Stony Athi and Mbagathi catchments, covering 2553 km^2^ within the Athi River basin, spanning Nairobi, Machakos, Kajiado, and a small section of Kiambu counties (Fig. 1). The catchments are located within a region extending 10 km on both sides of the Standard Gauge Railway (SGR) corridor, encompassing areas such as the Ngong-Athi-Kaputiei-Nairobi National Park ecosystem. The area’s altitude ranges from 1482 to 2448 m above sea level, averaging 1680 m. The Mbagathi River originates from Athi Spring in the Kibiku forest, while the Stony Athi River flows from Isinya, joining the Mbagathi River before draining into the Athi River and eventually the Indian Ocean [48]. Aquifer yields exceed 7.2 m^3^/h, with over 70% of the area producing this average, and a central section yielding 18–72 m^3^/h [49]. The region is primarily in the upper midland agro-ecological zone with arid and semi-arid conditions [50]. The Stony Athi catchment features eutric vertisols, haplic acrisols, and chromic cambisols, while the Mbagathi catchment has a mix of eutric vertisols, luvic phaeozems, humic nitosols, chromic cambisols, and rhodic nitosols [51, 52].

The area is served by three main water providers: Nairobi Water, Mavoko Water and Sewerage Company (MAVWASCO), and Oloolaiser Water and Sewerage Company (OLWASCO). Nairobi Water primarily depends on the Tana Basin, while Mavoko Water sources some supply from Nolturesh springs in the Mt. Kilimanjaro aquifers. Over 50% of water used in the catchments comes from boreholes, local rivers, and rainwater harvesting, emphasizing the importance of local hydrology on water supply. Water sources in the three counties vary. In Nairobi, 65.47% of households primarily used tap/piped water, while 28.20% relied on vended water. Kajiado county residents predominantly used vended water (35.7%) and boreholes (30.78%). In Machakos, borehole water was the primary source for 34.5% of households, and vended water was used by 28.53% [45].

The total population of the catchments in 2019 was 1,212,181, comprising 502,293 in Nairobi, 583,389 in Kajiado, and 126,499 in Machakos [53]. The annual population growth rates were 3.87% in Nairobi, 5.5% in Kajiado, and 2.47% in Machakos, all exceeding Kenya's overall growth rate of approximately 2.26%. Kenya's growth rate is expected to decline further to 1.78% by 2030, 1.03% by 2050, and 0.72% by 2063 [54]. The majority of the total population (84%) of the study area live in the urban areas [55].

### Methodology

The study used the Water Evaluation and Planning (WEAP) system to model water supply and demand, considering climate change, demographics, and land use [56–58]. Developed by the Stockholm Environment Institute (SEI), WEAP is a Decision Support System (DSS) based on the water balance concept, integrating supply, demand, water quality, and ecosystem preservation [59]. It requires data on climate, land use, Digital Elevation Model (DEM), water sources, and water uses.

- Climate data

Satellite-derived climate data were validated against ground observations due to limited in-situ data. Historical rainfall was analyzed using CHIRPS monthly data at a 5 km resolution (https://www.chc.ucsb.edu/data), and temperature using ERA5 monthly data at a 31 km resolution (https://www.ecmwf.int/en/forecasts/datasets/reanalysis-datasets/era5), both covering 1981–2019. Climate change scenarios (2021–2063) used CORDEX Regional Climate Models, specifically the CCCma and ICHEC models, chosen for their 77% correlation with observed data. Mann–Kendall trend analysis assessed historical and projected temperature and rainfall trends at a 95% confidence level.

- LULC analysis

Landsat 7 ETM (2000), Landsat 5 TM (2010), and Landsat 8 ETM (2019) satellite images, each at 30 m resolution, were used for Land Use and Land Cover (LULC) analysis. Sourced from the USGS Global Visualization Viewer (GloVis) (https://earthexplorer.usgs.gov/), the analysis employed remote sensing and GIS techniques. Images used were acquired between January and March, during the dry season with minimal cloud cover.

- Digital elevation model (DEM)

The Shuttle Radar Topography Mission (SRTM) DEM at 30 m resolution was downloaded from USGS GloVis (https://earthexplorer.usgs.gov/) and used to delineate the Mbagathi and Stony-Athi river catchments in the ArcGIS software.

- Water supply

Data on water sources and usage were collected from the 1999, 2009, and 2019 census reports [53] and household surveys in 2019. Using Yamane's (1967) [60] formula, a target sample of 384 households was estimated, with 305 (79.43%) questionnaires administered. Household heads or the next older person (18 +) were interviewed. ArcGIS was used to randomly select households, and the ArcGIS Collect mobile app was used for navigation. Informed consent was obtained from all respondents prior to administering the questionnaires. Participants were clearly informed about the purpose of the study and asked if they were willing to participate; the interviews proceeded only with their explicit consent. Data were captured using Open Data Kit (ODK). The questionnaire gathered information on water sources, usage, and socio-economic characteristics, analyzed using SPSS. Additional data on monthly water supply volumes, sources, and storage capacities were collected from Mavoko Water and Sewerage Company (MAVWASCO), Oloolaiser Water and Sewerage Company (OLWASCO), and Nairobi Water.

- Water demand

In the WEAP model, the main water uses (domestic, livestock, irrigation, commercial, and industrial) were designated as demand sites, each with a specific priority. Domestic demand had the highest priority, followed by livestock, irrigation, and lastly, commercial and industrial demands [61].

i) Domestic demand_human population (1999,** 2009**,** 2019)**

Human population data from census reports [53] were used to estimate domestic demand. Projected data was obtained from the World Population Review [55] and World Population Prospects 2024 [54]. Per capita water use estimates were from the Manual for Water Supply in Kenya within the National Water Master Plan 2030 [39]. Urban water use was averaged from high, medium, and low-class housing, while rural water use was estimated based on population density categories: high, medium, and low potential levels. Rural unit residential water demand was 92 L per person per day (l/p/d) in Nairobi, Mombasa, and Kisumu, 75 l/p/d in other urban centres, and 43 l/p/d in rural areas [39].

ii) Livestock demand_livestock population

Livestock population data came from the Kenya National Bureau of Statistics. Livestock water demand was set at 75 L per day per livestock unit (LU), where one LU equals one grade cattle, three native cattle, fifteen sheep/goats, five donkeys, or two camels [39, 62].

iii) Industrial water demand

To estimate industrial activity levels, the Kenya National Water Master Plan 2030 [39] method was used, calculating the activity level as a fraction of county firms to the total registered firms nationally. Industrial water demand was then determined as a percentage of urban domestic water demand, with high activity at 25%, medium at 15%, low at 5%, and non-activity at 0%.

iv) Commercial demand

Commercial water usage was set at 15% of urban and 10% of rural domestic demand, following Republic of Kenya (2013) [39] guidelines.

v) Agriculture demand

Land under cultivation, including both rain-fed and irrigated land, was determined through a combination of the LULC assessment and household survey data.

Land under cultivation, both rain-fed and irrigated, was determined using LULC assessment and household survey data.

Annual agricultural demand was based on the total irrigated area.

1 $$ {\text{Monthly}}\,{\text{ variation }}\,{\text{of}}\,{\text{ water}}\,{\text{ demand}} = \left( {{\text{Monthly }}\,{\text{K}}_{{\text{c}}} /{\text{Total}}\,{\text{ K}}_{{\text{c}}} } \right)*{1}00 $$

The water demand per crop was calculated using the following formula: -

2 $$ {\text{ET}}_{{\text{c}}} = {\text{ K}}_{{\text{c}}} *{\text{ET}}_{{\text{o}}} $$

where

ET_c_—crop evapotranspiration [mm/d]; K_c_—crop coefficient [dimensionless]; ET_o_—reference crop evapotranspiration [mm/d].and,

$$ {\text{Water }}\,{\text{Demand}}\,{\text{ per }}\,{\text{crop}} = {\text{ET}}_{{{\text{crop}}}} \times \, \left( {{1} + {\text{losses}}} \right) $$

ET_o_ was estimated using the FAO ET_o_ Calculator (http://www.fao.org/land-water/databases-and-software/eto-calculator/es/). The crop coefficient (K_c_), the ratio of crop ET_c_ to reference ET_o_, integrates crop height, crop-soil surface resistance, and albedo [63, 64]. FAO 56 was used to estimate K_c_ values for crops and vegetation in the catchment [63].

- Setting up of the WEAP model

**Fig. 2:**
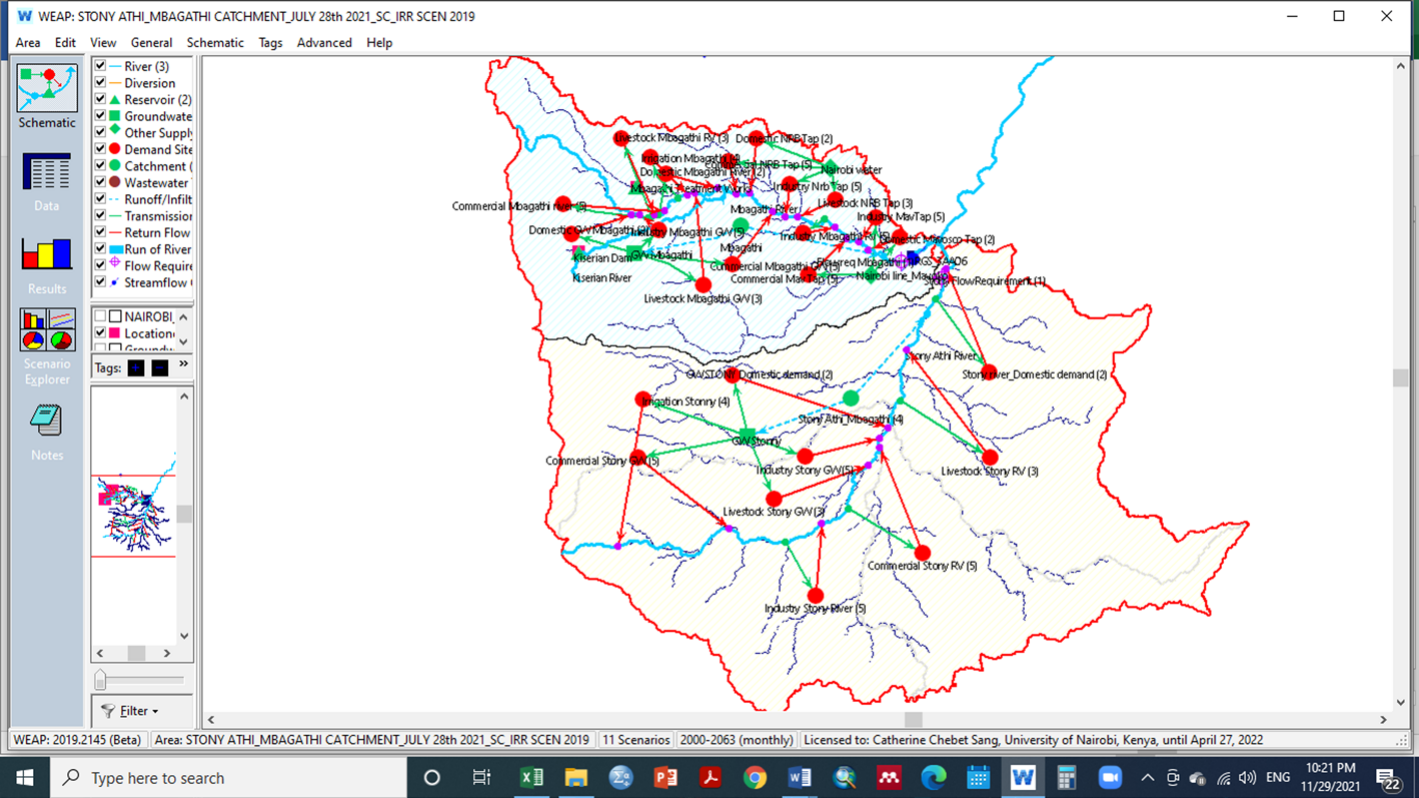
A schematic of the Mbagathi and Stony Athi catchments in the WEAP model

The WEAP model incorporated data on land use, climate, hydrology, DEM, human and livestock populations, irrigation, and commercial and industrial activities. All water supply sources and demand sites were identified and linked (see Fig. 2). The reference period, serving as the baseline in WEAP, was from 2000 to 2019.

- Calibration and validation of the WEAP model

Streamflow data from River Gauge Station (3AA06) for the Mbagathi River (2000–2006) were used to calibrate (2000–2002) and validate (2003–2006) the model [65]. Calibration involved adjusting model parameters and comparing simulated flows with observed data to reduce discrepancies between them [65]. Validation was carried out for an independent period to assess the model's predictive capability without further parameter adjustments. Model performance was assessed using Nash–Sutcliffe Efficiency (NSE), Percent Bias (PBIAS), Root Mean Standard Deviation Ratio (RSR), and coefficient of determination (R^2^) [66–68]. The results demonstrated strong model performance, with calibration values (NSE: 0.89, PBIAS: 8.19, RSR: 0.35, R^2^: 0.91) and validation values (NSE: 0.86, PBIAS: 15.42, RSR: 0.41, R^2^: 0.91), indicating that the model effectively captured observed hydrological patterns. Upon successful calibration and validation, the WEAP model was used to simulate multiple future scenarios, including climate change, population growth, and shifts in water demand.

### Scenario analysis

The WEAP model analysed scenarios including socio-economic development, demographic dynamics, and climate change to assess their impacts on water demand and supply (Table 1).

**Table 1:** Scenarios analysed in the WEAP model

Scenario	Description
Reference scenario (business as usual)	Assumes no interventions, with population, climate, and land use trends continuing as in the reference period
Projected population growth rate	Slower population growth of 1.58% per year (World Bank projections) versus 2.28% in the reference period [53]
Higher population growth rate	Growth rate of 2.76% per year, exceeding reference and World Bank projections, representing the highest projected growth
Higher urbanization rate	Urban population grows at 3.68% per year, reflecting higher rates than in the reference period [55]
Higher industrialization rate	Industrial growth of 4% per annum, surpassing the reference period's 3.32% [39]
Expansion of irrigation	Simulates maximum irrigation expansion for the Athi catchment by 2030, remaining constant until 2063 [39]
Climate change scenarios	**RCP 4.5**: Moderate impacts, baseline scenario [69, 70] **RCP 8.5**: Worst-case scenario with severe climate effects [71]

## Results and interpretation

### Historical climate, LULC, surface runoff, and water demand characteristics

The baseline period for climate analysis was 1981–2019, while the baseline (reference period) for the WEAP model was 2000–2019.

#### Climate

During the baseline period (1981–2019), mean annual temperatures increased by 0.89 °C, 0.88 °C, and 0.86 °C at Athi River, Ngong, and Kisaju stations, respectively, with Ngong showing the highest rise. Trend analysis revealed a statistically significant increase in temperatures at all stations (*p* = 0.00). Mean monthly temperatures ranged from 17.46 °C in July to 21.53 °C in March, with an average increase of 0.88 °C over the period. The average annual temperature rise was 0.02 °C per year. Rainfall in the catchments (1981–2019) shows an increasing trend, highest in Ngong, but the rise is statistically insignificant (p-values: 0.49 for Athi, 0.28 for Ngong, 0.32 for Kisaju). The average annual total rainfall increased between 1981 and 2019, though the trend was insignificant (*p*-value: 0.61). Driest years, linked to severe droughts, occurred in 1984, 1987, 2000, 2005, 2008–2009, and 2017 [72–74]. Rainfall has a bimodal pattern with peaks in April and November, showing decreases in DJF and MAM seasons and increases in JJA and SON, but trends were insignificant (*p*-values > 0.05). Coefficient of variation indicates high variability across all seasons: MAM (38%), DJF (48%), SON (33%), and JJA (44%) [75].

Water balance analysis using the WEAP model shows evapotranspiration accounts for 73% of total rainfall in the catchments, followed by surface runoff (19%) and infiltration (7%). Total annual evapotranspiration decreased from 1801.10 Million Cubic Metres (MCM) in 2000 to 1793.01 MCM in 2019. Surface runoff increased by 13.16% from 2000 to 2019, while infiltration declined slightly by 0.45 MCM. Monthly average streamflow ranged from 1.10 m^3^/s in July to 13.81 m^3^/s in November in 2000, with a mean of 5.01 m^3^/s and a standard deviation of 4.1 m^3^/s. In 2019, streamflow ranged from 1.67 m^3^/s in July to 13.14 m^3^/s in April, with a mean of 6.16 m^3^/s and a standard deviation of 3.67 m^3^/s.

#### Land use land cover (LULC) changes

Between 2000 and 2019, the Mbagathi-Stony Athi catchments were primarily characterized by shrublands, followed by bareland, grasslands, and croplands. During this period, built-up areas increased by 53.67%, shrublands by 36.63%, and forests by 2.03%. There were significant decreases in wetlands (− 99.32%), bareland (− 63.42%), grassland (− 39.52%), water bodies (− 39.27%), and cropland (− 8.85%). Grasslands and water bodies increased from 2000 to 2010 but decreased from 2010 to 2019, while forest cover decreased in the first decade and increased in the second. Other land use classes showed consistent trends throughout the study period (Table 2).

**Table 2:** LULC of the Mbagathi-Stony Athi Catchments for 2000–2019

	LULC (km^2^)			LULC CHANGE (km^2^)		
	2000	2010	2019	2000–2010	2010–2019	2000–2019
Shrub land	1357.23	1367.75	1854.43	+ 10.52	+ 486.68	+ 497.2
Bare land	616.34	589.99	225.43	− 26.35	− 364.56	− 390.91
Grassland	269.35	288.38	162.92	+ 19.03	− 125.46	− 106.43
Cropland	194.88	184.77	177.64	− 10.11	− 7.13	− 17.24
Forest	51.69	46.71	52.74	− 4.98	+ 6.03	+ 1.05
Built up	51.14	59.9	78.58	+ 8.76	+ 18.68	+ 27.44
Wetland	10.26	9.57	0.07	− 0.69	− 9.50	− 10.19
Water	2.19	6.04	1.33	+ 3.85	− 4.71	− 0.86
Total	2553.00	2553.00	2553.00	–	–	–

#### The impacts of LULC and climate on water resources (supply) for the reference period (2000–2019)

**Fig. 3:**
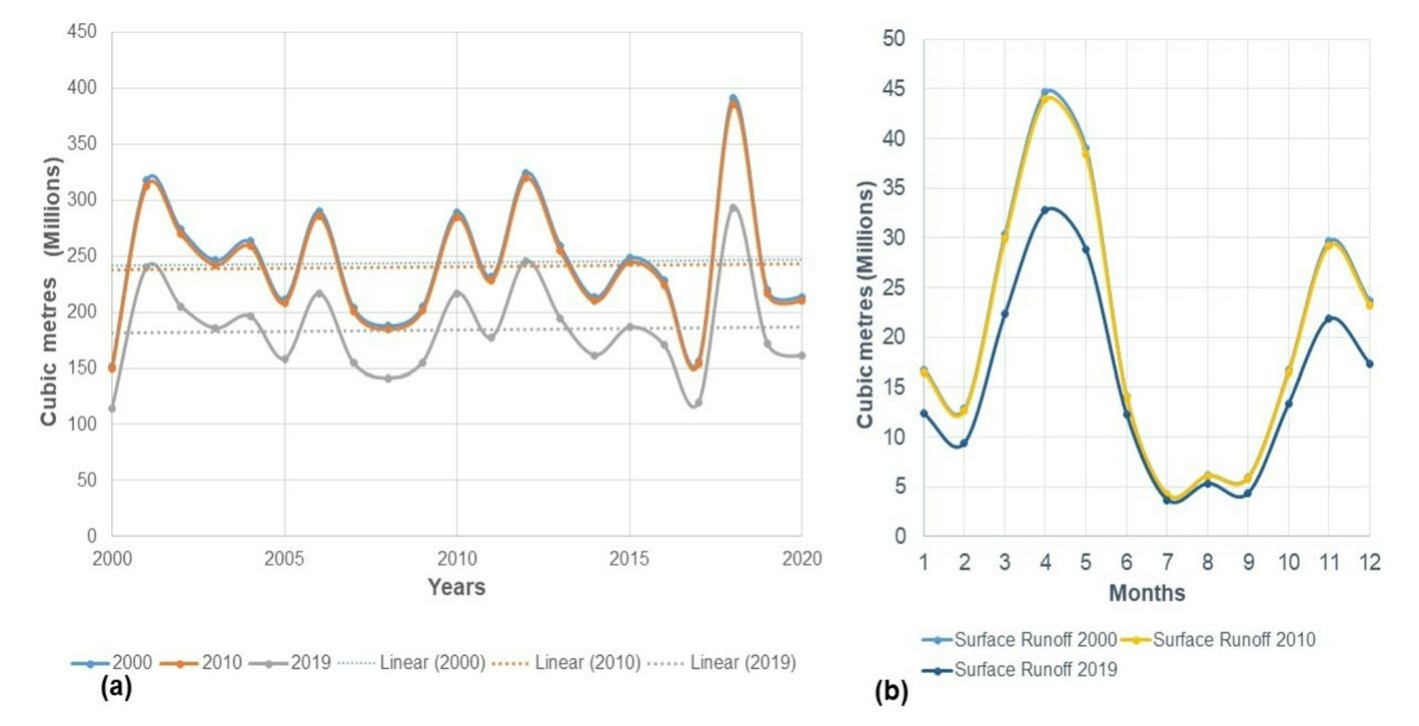
**a** The surface runoff for the Mbagathi––Stony Athi catchments under the 2000, 2010 and 2019 LULC regimes **b** Average monthly surface runoff reference scenario (2000–2019)

We assessed the impacts of land use and land cover (LULC) changes on surface runoff, evapotranspiration, and groundwater recharge within the WEAP model for the years 2000, 2010, and 2019, keeping climate factors constant. The results showed significant effects of LULC changes on surface water dynamics. The 2000 LULC had the highest annual surface runoff, followed by 2010, with 2019 having the least (Fig. 3a). The year 2000 had the lowest runoff in all LULC regimes, coinciding with a severe drought in Kenya [72, 76]. Despite increases in surface runoff during the reference period, statistical evidence for a significant trend was insufficient. The coefficient of variation (CV) for surface runoff was 23%, indicating moderate variability [75]. Average annual surface runoff increased by 31.50% from 2000 to 2019. Total surface runoff (2000–2019) was 4974.58 MCM under the 2000 LULC, 4839.16 MCM under the 2010 LULC, and 3705.59 MCM under the 2019 LULC. The increase in shrublands and decrease in bare land between 2000 and 2019 contributed to a 25.51% decline in surface runoff, highlighting the crucial role of land use changes in surface runoff dynamics.

The 2000 LULC consistently produced the highest average monthly runoff, followed by the 2010 LULC, with the 2019 LULC generating the least runoff (Fig. 3b). This trend is attributed to the higher extent of bare land in 2000, which decreased in 2010 and was lowest in 2019, contributing to increased runoff in 2000.

**Fig. 4:**
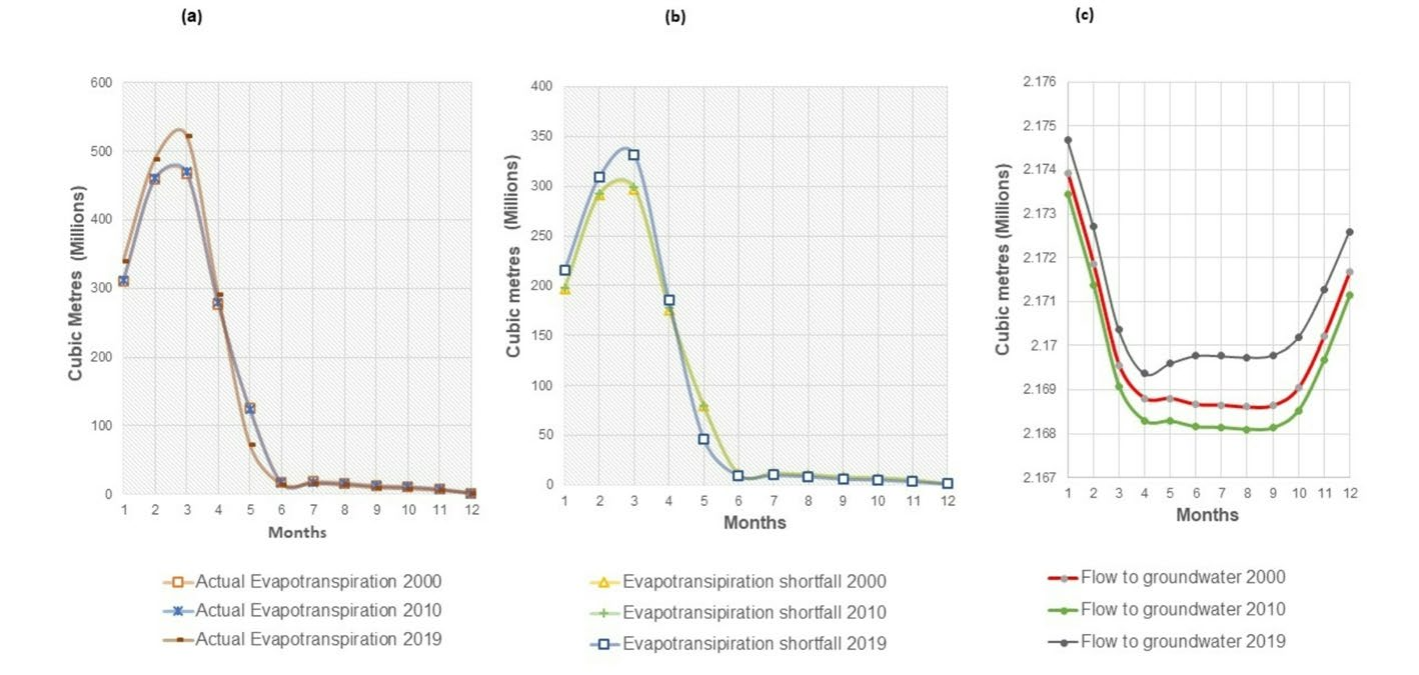
**a** Average monthly actual evapotranspiration and **b** evapotranspiration shortfall under the 2000, 2010 and 2019 LULC regimes during the reference period 2000–2019** c** Average monthly flow to groundwater during the reference period 2000–2019 under the 2000, 2010 and 2019 LULCs (1–12 = January–December)

The WEAP model simulation showed that the 2019 LULC had the highest average monthly evapotranspiration in December–April. The 2000 LULC had the highest in June–September, and the 2010 LULC peaked in October and November. March had the highest evapotranspiration for all regimes, coinciding with the highest mean temperature (Fig. 4a).

Total annual evapotranspiration increased by 3.55% from 2000 to 2019, mainly due to increased shrublands and forest cover in 2019 (Table 2). The 2000 LULC’s high evapotranspiration from June to September is linked to extensive cropland during these months.

The evapotranspiration shortfall was highest from January to March, corresponding to high temperatures and low rainfall (Fig. 4b). The 2019 LULC regime showed the greatest shortfall due to increased shrublands and forests, which heightened water demand for transpiration and increased infiltration, reducing available water for evapotranspiration. Early-year evapotranspiration is influenced by natural vegetation, while mid-year is driven by cropland, with the 2000 LULC having the highest shortfall during the growing season due to extensive cropland.

The total annual flow to groundwater (infiltration) slightly increased by 0.04% under the 2019 LULC compared to the 2000 LULC due to increased forest cover and decreased bare land. The 2019 LULC had the highest average monthly groundwater flow, followed by the 2000 LULC, with the 2010 LULC showing the lowest flow (Fig. 4c). This increase under the 2019 LULC is attributed to greater vegetation cover, especially forests, which reduces surface runoff and enhances water infiltration and percolation.

Our simulation of actual runoff for 2000, 2010, and 2019, based on respective rainfall, temperature, and LULC conditions, revealed the highest surface runoff in 2010 (284.95 MCM), followed by 2019 (171.52 MCM), and the lowest in 2000 (151.57 MCM). The variation in runoff was mainly due to rainfall differences, with 434.04 mm in 2000, 630.54 mm in 2010, and 828.39 mm in 2019. LULC changes also influenced runoff: 2010 had higher runoff than 2019, despite higher rainfall in 2019, due to reduced forest cover in 2010 and increased shrub land in 2019 (Table 2). Additionally, an increase in water bodies between 2000 and 2010 was reflected in the simulated runoff.

#### Water demand during the reference period (2000–2019)

During the reference period (2000–2019), water demand consistently increased, totalling 607.87 MCM. Domestic use was the highest demand category at 45.35%, followed by irrigation (27.64%), livestock (13.17%), commercial (9.26%), and industrial (4.58%). Annual water demand rose from 17.28 MCM in 2000 to 42.77 MCM in 2019. Throughout this period, water demand consistently exceeded supply, leading to unmet demands. The total demand was 607.87 MCM, while the supply was 510.44 MCM, resulting in an unmet demand of 97.43 MCM (16% of total demand). Despite an increase in water supply, it could not keep pace with growing demand. Between 2000 and 2019, the annual supply, demand, and unmet demand increased by 171.64%, 147.56%, and 73%, respectively.

Water demand site coverage, representing the percentage of water needs met, consistently fell below 100%. Most demand sites had coverage rates above 90%, except for irrigation, which averaged 55–70% between April and October. The overall monthly average coverage for all sites was 92.59%, indicating significant water scarcity in the catchments. In terms of reliability, the demand sites showed the following results: livestock 89.28%, domestic 89.06%, industry 82.29%, commercial 81.92%, and irrigation 70.83%, indicating water deficits and scarcity. The Nairobi Water and Sewerage Company meets international standards (> 80%) with 82% coverage, while MAVWASCO and OLWASCO fall below at 39% and 55%, respectively (38). Nairobi's higher coverage is due to sourcing water from the Tana basin, whereas MAVWASCO and OLWASCO rely more on local sources. High Non-Revenue Water (NRW) in all providers exacerbates water scarcity and highlights the need for better management [77].

In a nutshell, the Mbagathi-Stony Athi catchments saw significant temperature increases and land use changes during the reference period. Mean annual temperatures rose significantly at all stations, while rainfall increased slightly but with high variability and no significant trend. Droughts impacted the hydrological system. Land use shifts, such as more built-up areas and reduced natural surfaces, altered surface runoff and exacerbated water deficits. These disruptions affected groundwater and surface water availability. Despite rising water demand, especially for irrigation, supply did not keep pace, worsening water scarcity. Effective water resource management is essential to address these challenges.

### Projected climate and water demand scenarios (2021–2063)

#### Projected climate

Projections under RCP 4.5 and RCP 8.5 indicate rising mean annual temperatures for the future. Significant increases are forecasted, with p-values of 0.0000 for all stations in both scenarios. From 2021 to 2063, RCP 4.5 predicts increases averaging 0.89 °C, 1.39 °C, and 1.30 °C for Athi, Ngong, and Kisaju, respectively, compared to baseline (1981–2019) temperatures of 19.45 °C, 20.10 °C, and 19.38 °C. RCP 8.5 forecasts average increases of 1.28 °C, 1.81 °C, and 1.71 °C for the same stations. Overall, the mean annual temperature is projected to rise by 1.70 °C under RCP 4.5 and 1.75 °C under RCP 8.5 from 2021 to 2063, with an annual rate of + 0.04 °C per year under both scenarios.

Rainfall is projected to increase further under both RCPs 4.5 and 8.5, with RCP 8.5 predicting higher totals than RCP 4.5. Trend analysis shows a significant increasing trend for all stations under both scenarios, with* p*-values of 0.00, 0.01, and 0.03 for RCP 4.5, and 0.00 for all stations under RCP 8.5. Baseline annual rainfall was 615.45 mm for Athi, 838.56 mm for Ngong, and 643.08 mm for Kisaju. For the period 2021–2063, projected annual rainfall is 790.30 mm, 894.62 mm, and 663.10 mm under RCP 4.5, and 854.61 mm, 969.20 mm, and 715.70 mm under RCP 8.5 for Athi, Ngong, and Kisaju, respectively. Overall, rainfall is expected to increase by 12.43% under RCP 4.5 and 21.02% under RCP 8.5 compared to the baseline average of 699.03 mm. Annual rainfall is projected to increase from 681.88 mm in 2021 to 737.64 mm in 2063 under RCP 4.5, and from 758.79 mm to 1048.72 mm under RCP 8.5. Future rainfall patterns will remain bimodal with peaks in April and November, similar to the baseline period. Ngong is projected to receive the most rainfall, with higher peaks under RCP 8.5 compared to RCP 4.5. April will remain the wettest month, and July the driest.

#### The projected impacts of climate change on water resources/supply

##### Surface runoff

**Fig. 5:**
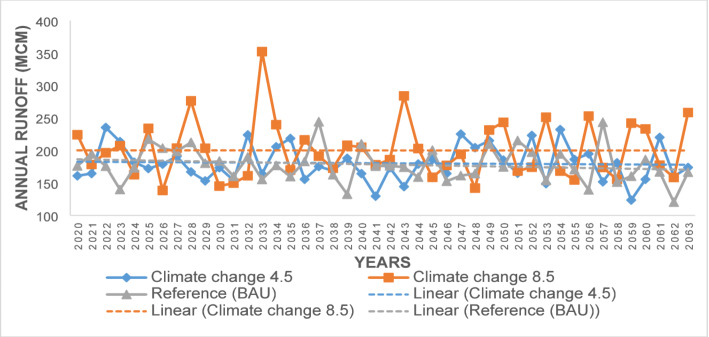
Projected surface runoff under RCP 4.5 and 8.5

Comparing surface runoff from the reference period (2000–2019) with projections under the reference scenario (Business As Usual) and climate change scenarios (RCPs 4.5 and 8.5) reveals diverse future trends (Fig. 5). During the reference period (2000–2019), the mean annual total surface runoff was 184.28 MCM. Projections for the future scenarios are 178.31 MCM under the reference scenario, 180.30 MCM under RCP 4.5, and 200.04 MCM under RCP 8.5. These projections indicate that RCP 4.5 and the reference scenario will have lower runoff, while RCP 8.5 will produce higher runoff compared to the reference period. For the near future (2020–2039), mean annual runoff is expected to decrease by 1.76% under the reference scenario and by 1.09% under RCP 4.5, while it will increase by 9.38% under RCP 8.5. Looking ahead to the period 2040–2063, a decrease of 4.47% and 3.05% in runoff is projected under the reference (BAU) and RCP 4.5 scenarios, respectively, whereas an increase of 7.86% is expected under RCP 8.5. Surface runoff under the reference and RCP 4.5 scenarios will show lower variability, with coefficients of variation (CV) of 15% each, while RCP 8.5 will have moderate variability, with a CV of 22%.

**Fig. 6:**
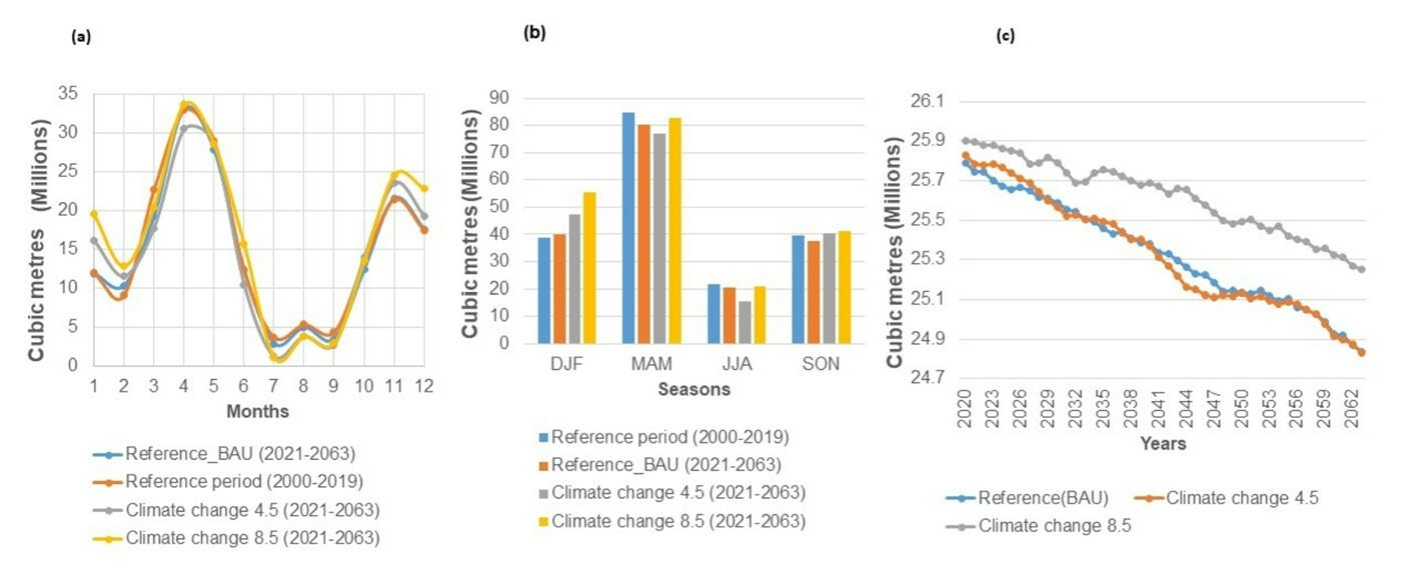
**a** Average monthly and **b** seasonal surface runoff in the reference period and climate change RCP 4.5 and 8.5 scenario **c** Flow to ground water under the reference, climate change RCP 4.5 and 8.5 scenarios

The findings reveal an increase in monthly average surface runoff under RCP 8.5 for most months, while runoff will generally be lower under RCP 4.5 and the reference scenarios. Minimum flows under all future scenarios (RCP 4.5, 8.5, and reference) are projected to be lower than those during the reference period. The reference period showed the least runoff from November to February and the highest in March, July, August, and September as compared to all the future scenarios (Fig. 6a). Seasonally, the reference period had the highest runoff in MAM and the lowest in JJA, with this pattern anticipated to persist in the future under all scenarios (Fig. 6b).

##### Flow to groundwater (infiltration)

The projected flow to groundwater shows a slight decrease in the future model (Fig. 6c). Total annual flow is expected to decrease by − 3.70% under the reference scenario, − 3.87% under RCP 4.5, and − 2.50% under RCP 8.5. Throughout 2020–2063, RCP 8.5 will have greater flow to groundwater compared to RCP 4.5 and the reference scenarios. The average monthly groundwater flow during the reference period was 2.17 MCM. Projections indicate future decreases: 2.1109 MCM under the reference scenario, 2.1106 MCM under RCP 4.5, and 2.13 MCM under RCP 8.5. Notably, RCP 8.5 will exhibit higher groundwater flow than the reference and RCP 4.5 scenarios.

##### Potential evapotranspiration

**Fig. 7:**
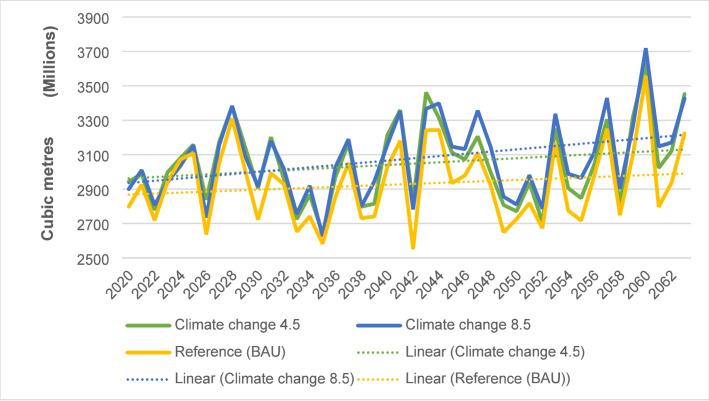
Annual potential evapotranspiration under the reference, RCP 4.5 and 8.5 scenarios

Potential evapotranspiration is projected to increase in the future under the reference, RCP 4.5, and RCP 8.5 scenarios (Fig. 7). Precisely, it is estimated to rise by 23.39% in the reference scenario, 16.44% in RCP 4.5, and 11.19% in RCP 8.5 during 2020–2063. The projected monthly average potential evapotranspiration will exceed reference period levels throughout the year, with more pronounced variations in the first four months. On average, monthly potential evapotranspiration is anticipated to increase by 0.36% in the reference scenario, 4.38% in RCP 4.5, and 5.38% in RCP 8.5 compared to the reference period.

#### The future water demand under various scenarios

An assessment of water demand impacts under various scenarios reveals diverse effects (Table 3). All future scenarios predict higher mean annual water demand compared to the reference period. The high urbanization scenario shows the most significant increase, with a 184% rise, highlighting the pressure from urban expansion. Both climate change scenarios (RCP 4.5 and 8.5) and the reference scenario predict a 134% increase in annual water demand. The population growth rate scenario projects the lowest water demand among those evaluated.

**Table 3:** Mean annual water demand (MCM)

Scenarios	Mean annual water demand (cubic meters)	Deviation from the reference period	Deviation from the business as usual scenario
Reference period (2000–2019)	30.39	0	− 40.64
Reference (BAU)	71.04	+ 40.64	0
Climate change 4.5	71.04	+ 40.64	0
Climate change RCP 8.5	71.04	+ 40.64	0
Expansion in irrigation	77.54	+ 47.15	+ 6.50
Higher industrialization rate	71.35	+ 40.96	+ 0.32
Higher population growth rate	75.79	+ 45.40	+ 4.76
Higher urbanization growth rate	86.25	+ 55.86	+ 15.22
Population growth rate projected	63.82	+ 33.42	− 7.22

**Fig. 8:**
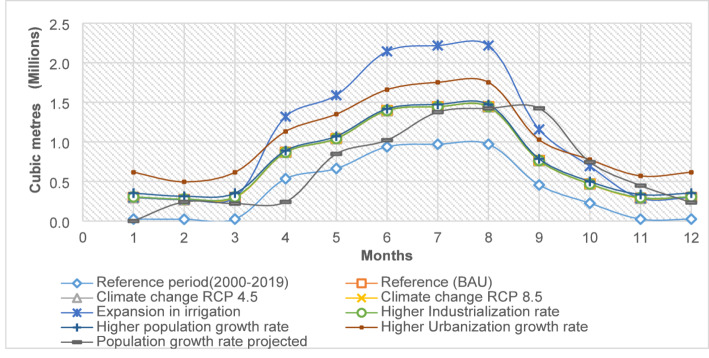
The projected average monthly unmet demands under various scenarios

The data showed significant unmet water demand in June, July, and August across all scenarios (Fig. 8), aligning with the lowest rainfall and high crop water demand, highlighting a critical shortage during key agricultural periods. Conversely, January, February, and March had lower unmet demands due to reduced water needs before the planting season in April. Unmet demands from October to December were also low, as this period is part of the wet season with increased water availability, and reduced water needs as most crops approach harvest.

The results showed variations in average monthly unmet demands across future scenarios, all exceeding reference period levels. The “expansion in irrigation” scenario had the highest unmet demands, increasing by 162.47% compared to the reference period. This was followed by “higher urbanization” at 153.78%, “higher population growth rate” at 91.12%, and “higher industrialization rate” at 83.02%. Both the “reference” and “climate change” (RCP 4.5 and 8.5) scenarios had a similar impact with an 82.08% increase. The least impact was from the “projected population growth rate,” with a 73.72% increase (Table 4).

**Table 4:** Average total annual unmet demands under various scenarios

	Total (MCM)	% increase from the reference period
Reference period (2000–2019)	4.87	
Reference (BAU)	8.87	82.08
Climate change RCP 4.5	8.87	82.08
Climate change RCP 8.5	8.87	82.08
Expansion in irrigation	12.78	162.47
Higher industrialization rate	8.92	83.02
Higher population growth rate	9.31	91.12
Higher urbanization growth rate	12.36	153.78
Population growth rate projected	8.46	73.72

The mean reliability of demand sites, representing the percentage of months with fully satisfied demand, varied across scenarios. The highest reliability was projected for the “projected growth rate” scenario at 86.24%, followed by “higher industrialization rate” (85.50%), “climate change scenarios” (85.49%), “expansion in irrigation” (85.49%), “reference” (85.49%), and “higher population growth rate” (84.40%). The lowest reliability was expected in the “higher urbanization growth rate” scenario at 82.25%. All scenarios show mean reliability below 90%, indicating potential water shortages and concerns about meeting water demands in the catchments.

The mean monthly coverage of demand sites is projected to be lower than the reference period across all scenarios, indicating challenges in meeting water needs year-round. Most scenarios show reduced coverage compared to the reference period, except for the “expanded irrigation” scenario, where livestock demand sites have higher coverage. The “higher urbanization growth rate” scenario has the lowest monthly demand site coverage, highlighting the difficulties in satisfying water needs in a rapidly urbanizing environment (Table 5).

**Table 5:** Average monthly demand sites’ coverage for the different demand sites under various scenarios

Scenarios	Average demand site coverage (%)					Deviation from the reference scenario (%)				
	Commercial	Domestic	Industry	Irrigation	Livestock	Commercial	Domestic	Industry	Irrigation	Livestock
Climate change RCP 8.5	79.42	94.94	79.42	78.92	85.20	− 15.17	− 2.69	− 15.53	2.64	− 14.31
Expansion in irrigation	79.42	94.94	79.42	75.92	100.00	− 15.17	− 2.69	− 15.53	− 0.36	+ 0.49
Higher Industrialization rate	79.33	94.94	79.33	79.17	85.20	− 15.26	− 2.69	− 15.62	+ 2.89	− 14.31
Higher Urbanization growth rate	72.81	90.86	73.24	79.95	83.23	− 21.78	− 6.77	− 21.71	+ 3.67	− 16.28
Higher population growth rate	77.58	93.64	77.58	79.80	84.74	− 17.01	− 3.99	− 17.37	+ 3.52	− 14.77
Population growth rate projected	81.73	96.70	81.73	77.66	86.68	− 12.86	− 0.93	− 13.22	+ 1.38	− 12.83
Climate change RCP 4.5	79.42	94.94	79.42	78.92	85.20	− 15.17	− 2.69	− 15.53	+ 2.64	− 14.31
Reference (BAU)	79.42	94.94	79.42	78.92	85.20	− 15.17	− 2.69	− 15.53	+ 2.64	− 14.31
Reference period (2000–2019)	94.59	97.63	94.95	76.28	99.51	0.00	0.00	0.00	0.00	0.00

In summary, from 2021 to 2063, the Mbagathi-Stony Athi catchments are expected to face significant changes and challenges. Mean annual temperatures will rise significantly under both RCPs 4.5 and 8.5. Rainfall will increase, with higher levels under RCP 8.5, while surface runoff is projected to decrease under RCP 4.5 and the reference scenario, but increase under RCP 8.5. Groundwater flow is expected to decrease slightly across all scenarios, and potential evapotranspiration will increase, leading to greater water loss. Water demand will rise, notably in the “expansion in irrigation” scenario, causing significant unmet demand during crucial agricultural periods. These trends highlight the need for effective water management, climate adaptation, and sustainable urban planning to address increasing demand and climate impacts.

## Discussion

This paper evaluates water supply and demand trends and explores the impact of development, demographic, and climate change scenarios on water availability in the Mbagathi and Stony-Athi River catchments. It provides essential insights to support sustainable management and ensure water security in the Athi River basin.

### Impacts of climate, land use, and urbanization on water resources and water demand in a changing landscape

Impacts of climate, land use, and urbanization on water resources in the Mbagathi-Stony Athi catchments are clear. Analysis shows a significant temperature increase, with statistically insignificant rainfall trends expected to continue, aligning with previous studies (e.g. [78–80]). These changes affect surface runoff, evapotranspiration, and groundwater, challenging water supply systems and worsening scarcity, as noted by [81]. Increased temperatures raise evaporation rates, reducing water resources, particularly in drylands. Variability in precipitation further strains water systems [82]. Monthly surface runoff correlates with rainfall fluctuations, reflecting seasonal changes [78, 83].

Urbanization in Nairobi has significantly altered land use, converting natural and agricultural areas into built-up environments [23, 84–86]. This shift reduces natural surfaces and vegetation, increasing impermeable areas and disrupting the hydrological cycle. Changes in land use impact water resources: more bareland leads to higher runoff and less infiltration, while increased vegetation enhances evapotranspiration and infiltration, reducing runoff [87–90]. Although previous studies linked urbanization with decreased groundwater recharge (e.g., [91–93]), our study did not find a significant impact on recharge but did note increased actual evapotranspiration with urban expansion [92, 93]. Overall, urbanization and impervious surfaces contribute to reduced groundwater recharge, increased runoff, and higher evapotranspiration, emphasizing the role of land cover changes in influencing these patterns [94–96]. To address these challenges, enhancing tree planting and constructing dams for surface runoff storage are recommended. Increasing green spaces in urban areas will help check runoff and improve infiltration. These measures will also support climate change mitigation. Additionally, controlling urban sprawl through effective land use planning is crucial.

The predominant water demand in the catchment is domestic, driven by urbanization and high population density. This growth has led to increased water demand and deficits, consistent with global urbanization trends [97–99]. Water scarcity is exacerbated by land use changes, temperature shifts, and altered rainfall, which impact water supply. Additionally, land-use changes increase soil erosion, affecting water body sedimentation and pollution, thereby compromising water quality and availability [28].

### Future water supply and demand scenarios and their implications for water security in the Mbagathi-Stony Athi river catchments

Future projections indicate that surface runoff will increase under RCP 8.5 but decrease under both the'business as usual' (reference) and RCP 4.5 scenarios compared to the reference period. Higher temperatures across all climate scenarios are expected to increase evapotranspiration and slightly decrease groundwater replenishment [100]. Despite a slight increase in rainfall, water resources remain insufficient, as a significant proportion of precipitation is lost through evapotranspiration, leaving only 30% for surface runoff and infiltration. This inadequacy is exacerbated by the region's semi-arid nature, low baseline rainfall, and expected significant temperature rise.

Annual water demand is projected to increase in the future across all scenarios, including ‘business as usual’. Water deficits will also be greater in the future under all scenarios compared to the baseline period, with the most significant shortages projected under the ‘expansion of agriculture’ scenario, followed by the'higher population rate' scenario. Urbanization will have a greater impact on water demand than other factors, primarily affecting domestic water demand. Urbanization influences both water availability and demand, contributing substantially to water insecurity if appropriate intervention measures are not implemented [91, 101–104]. A higher population growth rate is expected to drive future water demand, consistent with studies showing a positive correlation between population growth and water demand [105]. Ayeni [101] affirmed that rapid population growth intensifies pressure on water resources. Additionally, rapid population growth increases food demand, necessitating the expansion of agricultural activities to meet these elevated food requirements [106, 107].

The ‘expansion in irrigation’ scenario indicates a rise in agricultural water demand, resulting in higher total and unmet demands compared to the ‘business as usual’ scenario. This expansion is likely to increase water scarcity in the region [107–110]. Rapid industrialization will also contribute to higher future water demand, though to a lesser extent [111]. Rising water demand poses challenges in balancing water supply and demand, leading to shortages [112, 113]. While our findings suggest no significant impact on water demand under climate change scenarios, prior studies have demonstrated that climate change can substantially affect water demand, particularly in the context of irrigation, where increased demand is expected [109, 114–116].

These challenges of water supply and demand, exacerbated by urbanization, industrialization, population growth, and expanded irrigation, significantly contribute to water scarcity in the region, mirroring observations from other parts of the world [91, 100, 104, 106, 109, 110, 117]. As global water demand rises, the pressure on available water resources increases, posing risks to their sustainable utilization [117].

### Insights for sustainable transitions

Our study examined water resource availability and consumption in semi-arid catchments facing rapid economic development, urbanization, population growth, and climate change. These regions often suffer severe droughts due to low rainfall and rising temperatures, leading to water scarcity from high demands and limited resources [39]. Scenario analysis highlights the need for a multifaceted approach to water security in rapidly developing drylands. The catchments and the broader SGR corridor have seen a steady increase in water demand, projected to rise further due to population growth, urbanization, industrialization, and expanded irrigation. Meanwhile, water resources are expected to continue declining due to climate change and anthropogenic activities affecting land use and land cover.

i) *Addressing the water crisis.* The Republic of Kenya’s 2013 report [39] highlighted a water demand-to-resource ratio exceeding 40% in the Athi basin. In the catchments, Nairobi Water meets international water coverage standards, while MAVWASCO and OLWASCO fall short [38]. Nairobi's higher coverage is due to sourcing water from the Tana basin, whereas MAVWASCO and OLWASCO rely mainly on local groundwater and surface water, making *inter-basin transfers an effective strategy* for addressing water deficits. Inefficient water distribution, coupled with high levels of non-revenue water (NRW), further exacerbates scarcity. Improving distribution management, maintaining pipelines, and eliminating unauthorized connections are vital for enhancing water availability. Savings from reduced NRW can fund infrastructure expansion to improve access. To address the water crisis in the catchments, efforts are underway to drill more boreholes, but *sustainable groundwater extraction* is essential to prevent overexploitation [91]. Exploring interventions such as *rainwater harvesting*, including the establishment of dams and reservoirs, can enhance water availability and help manage regional flooding [114], particularly in light of recent flooding reports across the country. Additionally, implementing *soil and water conservation practices* is crucial to reduce surface runoff, soil erosion, and sedimentation in water bodies.
ii) *Factoring in growth and development pressures*. The development of the SGR and related mega projects is expected to drive population growth, rapid urbanization, and expanded industrial activities, exacerbating the water crisis in the catchments and the broader SGR corridor. To tackle this, sustainable water resources management is crucial, emphasizing an integrated approach [118]. *Integrated Water Resources Management (IWRM)* considers social, economic, and environmental factors, involving stakeholders, and promoting sustainable water resource utilization through adaptive management techniques [119, 120]. IWRM requires coordinated efforts among various institutions to develop *cross-sectoral solutions* and implement adaptive strategies. Collaboration across sectors is essential for sustainable water use and addressing water security challenges [121, 122]. Integrated planning, which considers agriculture, energy, and industry, enhances efficiency, reduces conflicts, and identifies synergies, such as using treated industrial wastewater for agricultural irrigation. This approach is vital for ensuring water security, meeting growing demands, balancing sectoral interests, and equitable water allocation [123, 124].
iii) *Building the human capacity*. Empowering local residents with knowledge and skills for sustainable water and natural resource management is essential. Efficient water use across agriculture, industry, and households is critical for addressing water scarcity [125–127]. Precision agriculture, industrial water recycling, and household initiatives like low-flow fixtures and leak repairs significantly contribute to water conservation [128–131]. These measures collectively play a crucial role in minimizing water consumption and addressing water scarcity. Besides, *capacity building and education* are crucial for sustainable water management, especially in addressing climate change [132, 133]. Continuous education keeps water professionals updated on modern practices and tools [134, 135], while policymakers need a solid grasp of water issues for effective legislation [133, 136]. Education helps professionals and policymakers understand climate-related water challenges and develop adaptive strategies [137]. Public education empowers communities to value water, reduce waste, and participate in conservation efforts [138, 139]. Training future young water professionals is essential for handling real-world situations, effective stakeholder communication, and fostering innovation in policy development and practical initiatives [135].
iv) *Strengthening planning and management processes.* Additionally, strengthening the integration of water resource assessment and accounting in development planning and implementation is crucial for comprehensive water security. Scenario analysis is vital for evaluating water security, considering factors like climate, demographics, and other uncertainties [140–142]. Catchment modelling anticipates events and facilitates strategic measures to address potential threats [143]. Decision Support Systems like the WEAP model allow simulations to plan for risks, ensuring sustainable water supply for human well-being, economic development, and ecological conservation [144–146]. However, effectiveness depends on comprehensive and accessible datasets, highlighting the importance of improving data collection and sharing among institutions. *Data and information sharing* are crucial for informed decision-making in water management [147]. Timely and accurate water resource data facilitate the assessment of water availability, quality, and distribution, aiding in addressing issues like scarcity, pollution, and climate change impacts. Open data platforms enhance water resource management by making data transparent and accessible to various stakeholders [148], fostering collaboration and supporting comprehensive strategies that consider climate, population, and land use changes. Reliable water data and open data initiatives are essential for tackling water security challenges and ensuring sustainable water management. Finally, *effective land use planning* and expanding green spaces in urban areas are also vital for water security. Effective land use planning prevents urban sprawl, balancing development with environmental preservation [149, 150]. Green spaces, like parks and gardens, act as natural sponges, reducing urban flooding and damage [151, 152]. Furthermore, they facilitate groundwater recharge, contribute to climate change mitigation by absorbing carbon dioxide, enhance air quality, and help alleviate the urban heat island effect [153–158]. This dual strategy enhances urban resilience and water security.
v) *Factoring in climate risks.* Our findings highlight the significant impact of climate change on water resources [159, 160], emphasizing the need for *adaptive strategies* to address shifting precipitation patterns and increased extreme weather events. Climate change exacerbates water security challenges, necessitating a transition to climate-resilient water management practices [137, 161]. *Nature-based solutions* such as rainwater harvesting, watershed management, wetland restoration, and afforestation enhance water retention, quality, and availability, contributing to climate resilience and long-term water security [162–164]. Sustainable land and water management, climate-resilient agriculture, and efficient irrigation systems are crucial for enhancing food security in drylands [127, 165, 166]. Ecosystem-based approaches, such as protecting and restoring wetlands and forests, are essential for maintaining water security [167, 168]. Prioritizing climate change adaptation measures, nature-based solutions, and sustainable land and water management practices can safeguard water resources' availability and sustainability, ensuring water security for future generations.
vi) *Embedding participatory nature-based approaches.* Furthermore, the research highlights the significance of *community engagement* and sustainable water use practices for addressing complex water challenges and ensuring responsible resource management. Community engagement entails involving local communities and stakeholders in the water-related decision-making process [169, 170]. This participatory approach considers regional hydrological dynamics, fosters ownership, and incorporates valuable local knowledge, especially from indigenous and traditional communities [170, 171]. *Integrating traditional wisdom* into modern water management enhances resilience against climate change [172]. Empowering communities leads to practical benefits, as community-led initiatives result in effective, equitable, and locally adapted solutions, building social capital and enhancing resilience to water-related challenges [173–176]. In arid and semi-arid regions like the studied catchments, these strategies are crucial for responsible water management, reducing environmental impacts, and enhancing climate resilience. Transitioning to sustainable water security is essential for ensuring the availability and quality of water for current and future generations. By embracing these practices, we can ensure water remains a sustainable and accessible resource for all.

## Limitations

The three most critical limitations of the study include limited streamflow data for calibrating and validating the WEAP model, reliance on satellite-derived climate data, and assumptions in estimating water demand, particularly for domestic and agricultural use. To address these constraints, proxy data from nearby catchments and regional datasets were utilized, and satellite data were validated against available ground observations to enhance reliability. Standardized demand estimates from national guidelines were supplemented with household survey data. While these remedies improve the model’s credibility under current circumstances, future studies should prioritize continuous hydrological monitoring, improved access to ground-based climate data, and localized water use audits for more accurate demand projections.

## Conclusion

The findings highlight a looming water scarcity crisis in the Mbagathi and Stony Athi catchments, driven by increasing water demand, land use changes, and climate variability. Projected increases in temperature, evapotranspiration, and urbanization will exacerbate water stress, with unmet demand rising significantly, particularly under irrigation expansion and urbanization scenarios. Surface runoff is expected to decline under the reference and RCP 4.5 scenarios but increase under RCP 8.5, indicating varied hydrological responses to climate change.

These results underscore the urgent need for integrated water resource management, incorporating sustainable land use planning, efficient water use, and climate adaptation strategies. The WEAP model proves valuable in scenario analysis, bridging data gaps and informing policy decisions. To ensure long-term water security, authorities must adopt proactive conservation measures, invest in resilient infrastructure, and engage communities in sustainable water management. By integrating these approaches, water security can be enhanced in the region and other semi-arid environments facing similar challenges.

While this study provides valuable insights, gaps remain in understanding groundwater dynamics, the role of nature-based solutions, and the socio-economic impacts of water scarcity. Future research should focus on these areas to strengthen adaptive strategies for sustainable water resource management.

## Data Availability

All relevant data supporting the findings of this study are included within the manuscript. Additional data can be made available by the corresponding author upon reasonable request.
